# PepFuNN: Novo Nordisk Open‐Source Toolkit to Enable Peptide in Silico Analysis

**DOI:** 10.1002/psc.3666

**Published:** 2025-01-07

**Authors:** Rodrigo Ochoa, Kristine Deibler

**Affiliations:** ^1^ Novo Nordisk A/S, Novo Nordisk Park Måløv Denmark; ^2^ Novo Nordisk Research Center Seattle, Novo Nordisk A/S Seattle Washington USA

## Abstract

We present PepFuNN, a new open‐source version of the PepFun package with functions to study the chemical space of peptide libraries and perform structure–activity relationship analyses. PepFuNN is a Python package comprising five modules to study peptides with natural amino acids and, in some cases, sequences with non‐natural amino acids based on the availability of a public monomer dictionary. The modules allow calculating physicochemical properties, performing similarity analysis using different peptide representations, clustering peptides using molecular fingerprints or calculated descriptors, designing peptide libraries based on specific requirements, and a module dedicated to extracting matched pairs from experimental campaigns to guide the selection of the most relevant mutations in design new rounds. The code and tutorials are available at https://github.com/novonordisk‐research/pepfunn.

## Graphical abstract

PepFuNN is a new open‐source version of the PepFun package with functions to study the chemical space of peptide libraries and perform structure–activity relationship analyses. It is a Python package comprising five modules to study peptides with natural amino acids and, in some cases, sequences with non‐natural amino acids based on the availability of a public monomer dictionary.

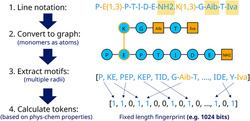

## 1 Introduction

Computational methods have significantly advanced several approaches toward rational peptide therapeutic design [1, 2]. Open‐sourced frameworks can accelerate the analyses of complex peptides, reducing human error while implementing everyday tasks around peptide in silico analysis [3, 4]. Most of the protocols are machine learning models built to accelerate the design of therapeutic peptides [5], with some focused on specific disease areas where peptides have played a significant role [6, 7]. With the rise of generative artificial intelligence (AI) protocols, new methodologies are continuously published to cover the design of peptides at different levels, from plain sequences to complex 3D structures [8, 9, 10]. Despite the accelerated progress on AI pipelines, the availability of tools to enable structure–activity relationship (SAR) analyses for peptides is still lacking compared to other therapeutic modalities like small molecules.

A combination of protein‐ and small molecule‐oriented tools is necessary to support the computational design and analysis of peptide libraries given their hybrid nature [11]. An example of an open‐source alternative to tackle this problem is the PepSeA tool that enables the multiple sequence alignments of peptides for SAR purposes [12], even in the presence of non‐canonical amino acids using specialized line notations like HELM [13]. The optimization of peptides using non‐canonical elements is a standard approach to enhance different peptide properties using classical virtual screening approaches [14] or through cheminformatics packages like pyPept, which allows the generation of 2D and 3D molecular representations of complex peptides for further in silico analysis [15].

PepFun is one of these open‐source tool alternatives, which embeds open bioinformatics and cheminformatics protocols to study peptide properties, run alignments, and provide functionalities to analyze structures and their interactions with molecular targets [16]. A second version of PepFun 2.0 was built as a Python package that additionally incorporates improved peptide similarity pipelines, peptide structure modification routines, estimation of amino acid‐based molecular descriptors, and some methods to study modified peptides based on a public monomer dictionary [17]. Here, we present PepFuNN, a new version of the PepFun package developed within the Novo Nordisk environment and highly focused on performing peptide SAR analysis. We provide details of the main modules, code workflows, and use cases where the tool can support peptide research projects and design campaigns.

## 2 Methods

### 2.1 Requirements

PepFuNN is a Python 3 package that can be installed locally based on the code instructions. PepFuNN requires external dependencies that can be installed automatically through PyPI. The main required package for the cheminformatics analysis is the RDKit (version 2023.03.3), available at https://rdkit.org/. For the bioinformatics part, different modules from BioPython are used (version 1.79) https://biopython.org/ [18]. The code includes a set of tests to verify the correct installation and primary usage of the package, as well as notebooks to follow the main functionalities.

PepFuNN contains five modules with different functionalities explained in Section 3. However, we highlight two methodologies for assessing the similarity of complex peptides and identifying peptide matched pairs to study hotspots and key mutations from bioactivity datasets.

### 2.2 Monomer‐Based Fingerprint Similarity

This methodology is a similarity approach relying on fingerprints built according to the monomer composition. The Morgan approach for small molecules inspires the generation of the fingerprints [19], but with the monomers playing the role of the atoms instead. The peptide is converted to a graph to capture additional bonds and connections to new chains made by residues belonging to the linear main sequence. The graph‐based representation is generated using the igraph module from Python [20]. With the graph, it is possible to represent complex peptides having multiple chains, cyclic structures, or the addition of non‐canonical elements. Internally, we use the BILN notation to generate the chemical representation [21]. Then, we go through the graph and split it into fragments of different radii (i.e., two or three consecutive monomers) in all the possible directions.

**FIGURE 1:**
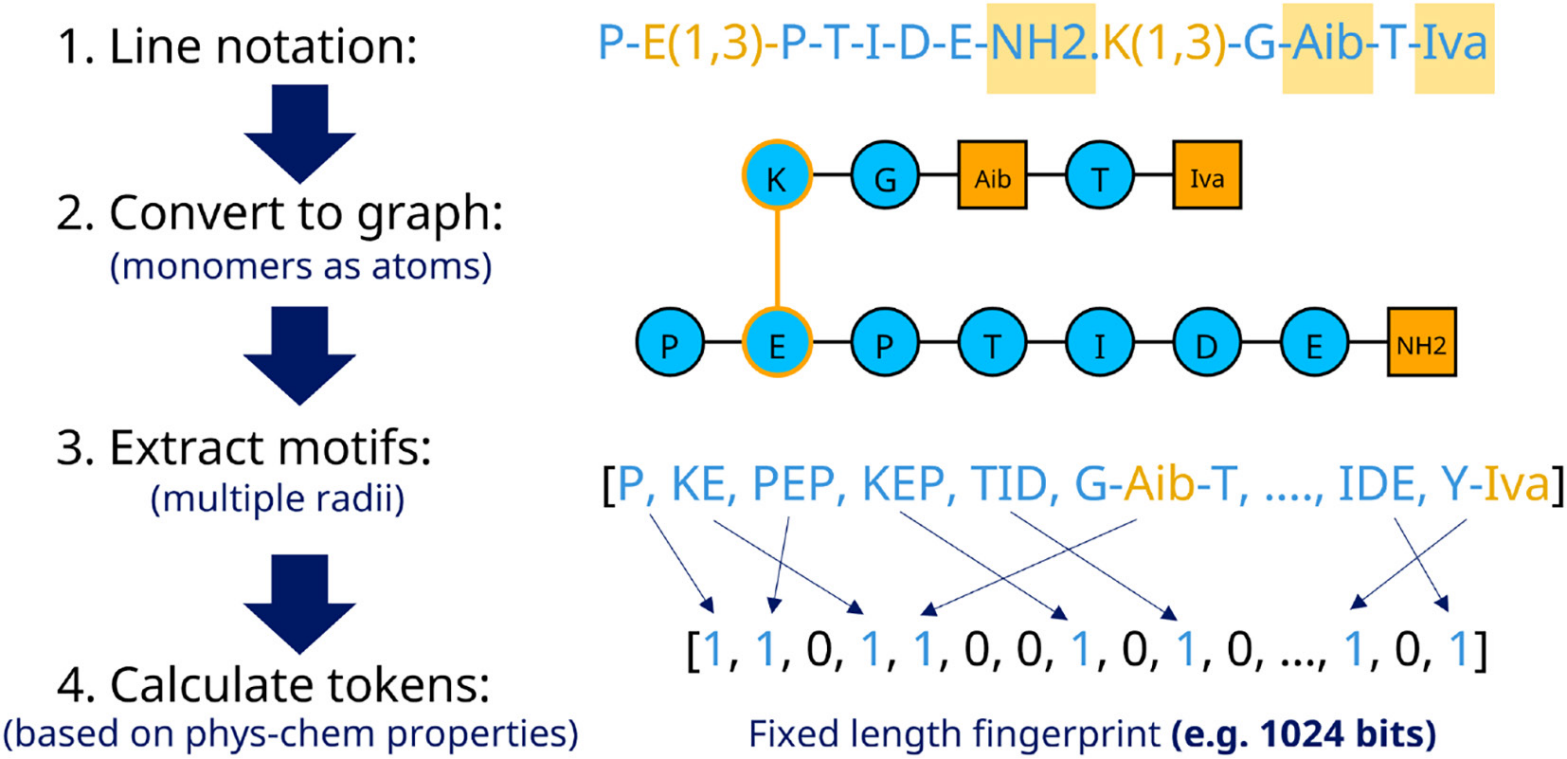
Summary of the methodology to build monomer‐based fingerprints for peptide similarity analysis. First, a line notation that is sensible to complex chemical structures is used to represent the peptides. In the example, we show a peptide with a C‐terminal cap, a second chain connected by a bond between the side chain of the glutamic acid (E) in the first chain with lysine (K) in the second chain, and two non‐canonical amino acids: Aib and Iva. The complex peptide is represented as a graph based on the previous instructions in the second step. Then, the graph is decomposed into fragments up to a defined radius (maximum of three consecutive monomers). Per each combination, a numerical token is generated based on a combination of physicochemical properties per monomer. In the final step, each token is converted into a bit that will be part of a fixed‐size fingerprint used to run similarity searches.

For each fragment, the physicochemical properties of the monomers are used to assign a specific code or token. For example, if we have the fragment glycine‐tyrosine‐histidine (GTH), we aggregate various properties by summing or multiplying their values per each monomer in the fragment. The properties considered are the number of heavy atoms, rotatable bonds, hydrogen bond donors and acceptors, heteroatoms, topological surface area, and molecular weight. In addition, the algorithm captures the frequency of the fragment within the peptide graph, which can be included or not by multiplying the numerical token by the number of times the fragment is present in the graph. Then, a hash code and a bit position are generated to assign a 1 or 0 in a fixed‐length fingerprint (i.e., 1024 or 2048 bits). The method generates a fingerprint per molecule, which can be used to run Tanimoto similarity searches by counting the number of matches and mismatches with the other fingerprints [22]. A summary of the whole approach is shown in Figure 1.

One crucial aspect of the method is that different fingerprints can generate bits in the same position due to similar physicochemical properties. This means that the fingerprint does not need to explore all the possible combinations of monomers, which is a typical exhaustive strategy using 2‐mer and 3‐mer counting vectors as descriptors in machine learning approaches [23]. A recommendation is to calculate all the fingerprints for a dataset of interest first and then quickly perform similarity searches using metrics like Tanimoto or other small molecule‐inspired comparison metrics.

### 2.3 Matched Pairs for Peptides

**FIGURE 2:**
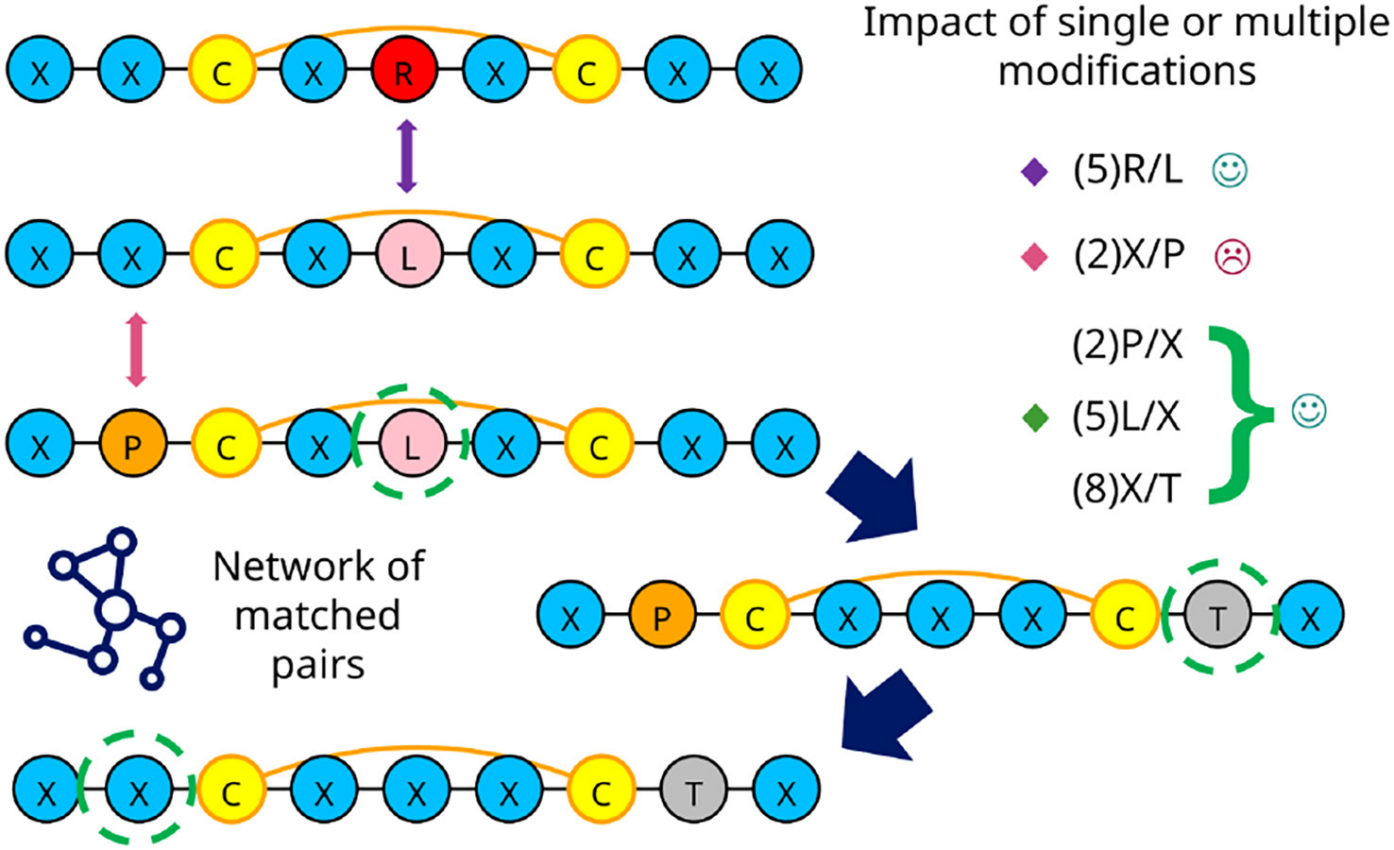
Schematic representation of the matched pairs analysis. A set of cyclic peptides are depicted with different single or multiple substitutions between the sequences. On the right are examples of mutations improving or decreasing a particular endpoint, which can be a potency assay within a design campaign round.

One strategy in small molecules to run structure–activity relationship (SAR) analysis is to identify matched molecular pairs (MMP) [24]. This cheminformatics methodology compares the properties of two molecules that differ by a single chemical transformation. The method is relevant to deeply exploring SAR data and collecting ideas for designing optimized molecules [25]. Similarly, the concept can be applied in the peptide field, where the transformation leads to substituting an amino acid or a non‐canonical element [26]. The method's effectiveness relies on how the peptide library is designed in the first place. Optimal libraries allow capturing the impact of a single or few modifications to generate better enumeration rules for future design rounds. With this analysis, a network of substitutions can be created to map the positive or negative effect of the changes about a desired endpoint, like a potency assay in a design campaign. A graphical summary of the approach is shown in Figure 2.

The method has been used before in a pharmaceutical context to optimize peptide ligands such as GPCR binders [27]. In PepFuNN, we provide two options to run the analysis: using reference sequences or not to guide the alignments between peptides. One advantage of using a reference is that it facilitates the enumeration of the peptide positions to identify better which specific position the change is occurring in and, consequently, annotate potential hotspots. If no reference is provided, the algorithm runs pairwise alignments between the sequences, with the option to open gaps. Here, the enumeration relies on how the algorithm matches the residues and can be helpful for more heterogeneous sequence datasets.

When analyzing peptide as ligands, the presence of gaps after aligning a series of analogs can be managed if there is a clear understanding of specific deletions or insertions in parts of the sequences. However, when no reference is provided, it is more challenging to control the generation of such gaps in large groups of sequences, posing difficulties in understanding the SAR based on the impact of substitutions in certain positions. Because of this, we recommend to provide a reference sequence when possible to better control the changes and enumeration during the SAR exploration.

## 3 Results and Discussion

### 3.1 Modules

**FIGURE 3:**
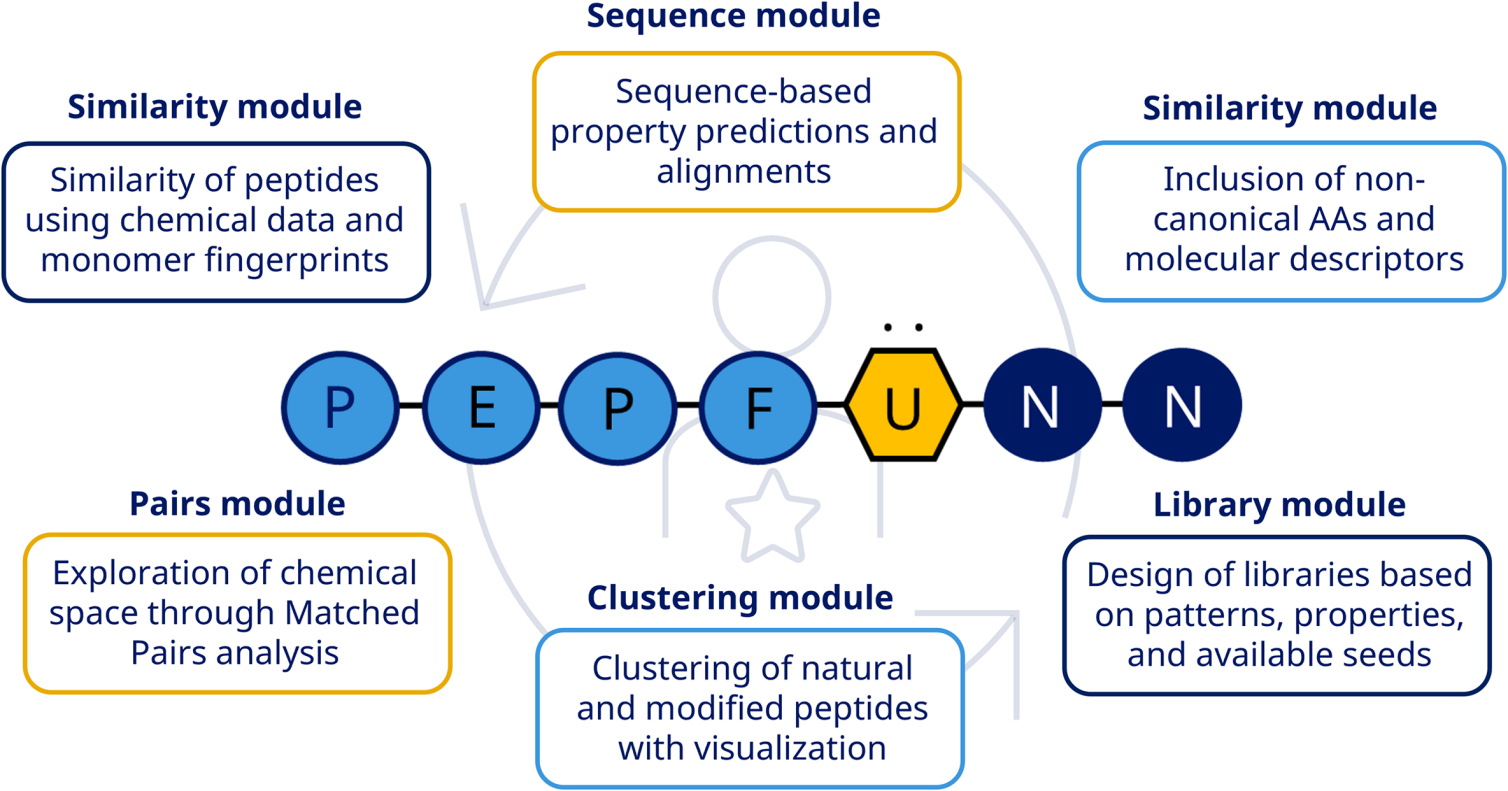
Overview of the modules included in PepFuNN with a brief description of the main functionalities.

PepFuNN contains five modules: Sequence, Similarity, Clustering, Pairs, and Library. These embed multiple classes to perform tasks like aligning sequences, calculating amino acid physicochemical properties, running clustering analysis, calculating molecular matched pairs, and designing peptide libraries based on different criteria. A graphical overview is shown in Figure 3. The following sections provide more specific details per module.

#### 3.1.1 Sequence Module

This module allows for calculating basic properties for peptide sequences using the FASTA format or the BILN notation. If the BILN notation contains non‐canonical residues, these are internally converted to their closest natural amino acid to run the functions with the natural peptide amino acid sequence as the input. The natural analogs are identified after running a fingerprint‐based similarity pipeline in RDKit between the non‐canonical residue and all the natural amino acids. The properties for the whole peptide sequence are calculated based on properties of natural amino acids available in metrics like the hydrophobicity Eisenberg scale [28]. The peptide net charges are estimated based on a list of pKa and pH values, and additional properties are obtained from the ProtParam package, including aromaticity, instability index, and statistics of the amino acid composition [29]. Additionally, a chemical representation using the SMILES format of the entire peptide can be used to calculate descriptors from the RDKit package, such as molecular weight, lipophilicity by the Crippen LogP [30], topological surface area (TPSA), among others.

The module contains a set of empirical rules and alerts about the peptides' potential solubility and synthesis issues. The rules describe violations by specific amino acid patterns, where the higher the number of violations, the lower the probability of validating the peptides experimentally [16, 31]. In Table 1, we describe the rules that were obtained from different online resources, including the peptide synthesis analysis tool from Thermo Fisher (online server), and the SolyPep server to generate sequences with aqueous solubility (online server). Overall, the rules are helpful to filter candidates and reduce the number of false positives during design phases.

**TABLE 1:** Description of the empirical rules and alerts to avoid potential solubility and synthesis experimental issues with peptides.

Rule/alert	Description
Solubility rule 1	Warning if the number of charged and/or of hydrophobic amino acids exceeds 45%
Solubility rule 2	Warning if the absolute total peptide charge at pH 7 is more than +1
Solubility rule 3	Warning if the number of glycines or prolines is more than one in the sequence
Solubility rule 4	Warning if the first or the last amino acid is charged
Solubility rule 5	Warning if any amino acid represents more than 25% of the total sequence
Synthesis rule 1	Warning if two prolines are consecutive
Synthesis rule 2	Warning if the motifs DG (aspartic acid and glycine) and DP (aspartic acid and proline) are present in the sequence. Two rules, one per motif
Synthesis rule 3	Warning if the sequences end with aparagine (N) or glutamine (Q) residues
Synthesis rule 4	Warning if there are charged residues every five amino acids
Synthesis rule 5	Warning if there are oxidation‐sensitive amino acids like methionine (M), cysteine (C), or tryptophan (W). Three rules, one per amino acid

Finally, a function to run online blast searches with a peptide of reference is available. The method was customized with parameters adjusted for matching peptide sequences [32].

#### 3.1.2 Similarity Module

In this module, we include various approaches for comparing peptide sequences having different lengths and, in some cases, containing non‐canonical amino acids. For the latest, we included a set of monomers based on the open HELM monomer dictionary (https://github.com/PistoiaHELM/HELMMonomerSets). A total of 322 monomers can be embedded with the BILN notation, where the monomers are separated by dashes and represented by the symbols stored in the dictionary. However, the user can add any new monomer by modifying the monomer SDF file, which is available in the data folder of the base code. By keeping the same monomer definition syntax and providing an abbreviation, the PepFuNN package can be re‐installed to allow using the monomer with the BILN notation.

We provide multiple configurations for the alignments using the peptide sequence or its chemical structure. We split the methods for sequences with the same or different lengths. In the case of peptides of the same size, the alignment score can be unweighted by considering only matches and mismatches or weighted by a similarity‐based scoring matrix pre‐calculated for all the monomers in the dictionary. The scoring matrix can be updated if new monomers are included or if different identity thresholds are required. For peptides with different lengths, we use the pairwise2 functionality from Biopython [18] with the same unweighted and weighted scenarios, where gaps can also be included. One requirement is to provide the sequences with the BILN notation to enable the similarity calculation for peptides containing non‐canonical amino acids that are described in the monomer dictionary. We provide a function to normalize the similarity scores between peptides A and B to quantify the similarity between the peptides. To achieve that, we use the following formula:

$$S_{\mathrm{AB}}=\frac{{\text{score}}_{\mathrm{AB}}}{\sqrt{{\text{score}}_{\mathrm{AA}}*{\text{score}}_{\mathrm{BB}}}}$$

where score_AB_ is the alignment score between the two peptides, and score_AA_ and score_BB_ are the alignment scores for each peptide with itself. Additionally, thanks to the RDKit package, we provide a function to run similarity calculations using the peptides Morgan fingerprints based on the Tanimoto coefficient [33]. A radius of four in the fingerprints is used by default to capture larger fragments found in repetitive structures like peptides.

The module includes a second class to generate descriptors for peptides based on pre‐calculated properties of the HELM monomer dataset. These properties are the molecular weight, topological polar surface area (TPSA), partition coefficient (LogP), and the number of rotatable bonds. A function calculates the autocorrelation of these properties to generate the descriptors using the Moran equation [23], which can be used to train machine learning models. A detailed explanation of the strategy is available in the PepFun 2.0 manuscript [17].

#### 3.1.3 Pairs Module

This module contains a class to run matched pairs analysis on peptides, as explained in Section 3.2. The basis is identifying subtle changes driving an assay to a specific goal (i.e., improving peptide candidates' potency in new design rounds) [26]. In the class, there are two ways of assigning the pairs. The first one uses a reference peptide to enumerate the positions to control better where the changes occur. The similarity threshold can also be modified to capture variants with several mutations concerning the reference. The second approach is to assign pairs without a reference and add gaps if necessary if the peptides have different sizes. This approach is the most challenging scenario in which to run SAR analysis but can be adapted depending on the project's needs. In both cases, the first step is to enumerate the positions for each peptide and then calculate all the pairs. The latest allows capturing, for example, the fold difference between the two activities using the ratio. The pairs consider directionality between the two compared molecules, where fold‐differences greater than one mean an improvement in the activity, and values below one have a detrimental effect. In addition, based on the frequency and number of simultaneous mutations, the user can rank those with higher chances of improving an observable for further peptide design runs.

#### 3.1.4 Clustering Module

With the chemical representation of the peptide, it is possible to calculate the similarity between molecules and use that to explore the chemical space for a set of peptides. Such distances can rely on structural differences using molecular fingerprints, calculating differences on physicochemical descriptors, or just using the amino acid sequence hamming distance [34]. The three methods are included in the class to cluster the molecules, and are named *simClustering*, *propClustering*, and *sequence_clustering*, respectively. In the case of fingerprint similarity (*simClustering*), functions to create *t*‐distributed stochastic neighbor embedding (t‐SNE) plots of the chemical space are available [35], with the possibility to color by the assigned cluster using the Butina algorithm [36]. It is also possible to plot principal component analysis (PCA) of the descriptors and clusters of the molecules based on the K‐means algorithm for the *propClustering* function. A specific example of this functionality is shown in Section 3.3.

#### 3.1.5 Library Module

Finally, this module allows the creation of peptide libraries restricted to natural amino acids by following rules based on amino acid patterns or by generating analogs of hits using random or suggested changes. These changes can be obtained from SAR exploration analysis, such as the peptide matched pairs approach. The method permits the generation of sequences using natural amino acids, which can be directly assigned or suggested based on their similar physicochemical properties like polarity or charge. In addition, extra filters can be activated to design sequences having distinct molecular weights to facilitate mass spectrometry deconvolution. Properties calculated in the Sequence module can be used to avoid highly hydrophobic or polar sequences and peptides with potential solubility or synthesis issues. Depending on the library's final goal, the user can customize the number of peptides and the possible rules. As in the previous class, we included an example showcasing the capabilities of the methodology in Section 3.3.

### 3.2 Workflows

The main goal of the PepFuNN package is to be used directly in Python scripts to run the main functionalities alone or in combination with other packages. Here, we provide three example workflows highlighting different functionalities from PepFuNN. However, for a more complete tutorial, please follow the notebooks provided in the code repository. The first example is to calculate the basic properties of a single linear peptide:from pepfunn.sequence import Sequencepep = Sequence("FNCREWCWN")netCharge=int(pep.netCharge)avgHydro=pep.avg_hydroisoPoint=pep.isoelectric_pointsol=pep.solubility_rules_failedsyn=pep.synthesis_rules_failedprint("Net charge: {:.2f}".format(netCharge))print("Average hydrophobicity: {:.2f}".format(avgHydro))print("Isolectric point: {:.2f}".format(isoPoint))print("Number of solubility rules failed: {:.2f}".format(sol))print("Number of synthesis rules failed: {:.2f}".format(syn))


The output to the previous snippet isNet charge: 0Average hydrophobicity: −1.44Isolectric point: 5.99Number of solubility rules failed: 1Number of synthesis rules failed: 1


When the sequence object is created, all the properties are calculated automatically. In the code snippet, we only capture five: net charge, average hydrophobicity, isoelectric point, and the number of failing solubility and synthesis rules. The code also includes notebooks where we exemplify additional properties and how to generate a full report with the empirical rules failing for the input sequence.

In the following snippet, we have a code fragment that allows alignments to be run in four different ways. The first is an alignment of two peptides of the same size that are scored by a similarity matrix. The second is the exact alignment, but the scoring is based on the Hamming distance. The third is the alignment of two peptides having different lengths, and the fourth one are two peptides with non‐canonical amino acids using the BILN notation:from pepfunn.similarity import Alignmentseq1="AFTGYW"seq2="AGTGYL"score1 = Alignment.align_samelen_matrix(seq1, seq2)score2 = Alignment.align_samelen_local(seq1, seq2)print(f"Score using a local matrix: {score1}")print(f"Score using the Hamming distance: {score2}")seq3="WWSEVNRAEF"seq4="KTEEISEVNIVAEF"score3, a1, a2 = Alignment.align_difflen_matrix(seq3, seq4, mode="unweighted")print(f"Score for peptides with different length: {score3}")print(f"Aligned sequence 1: {a1}")print(f"Aligned sequence 2: {a2}")seq5="K-Aib-M-P"seq6="S-A-Aib-P"score4, a3, a4 = Alignment.align_difflen_matrix(seq5, seq6, mode="weighted")print(f"Score for peptides with non-canonical residues: {score4}")


The obtained scores areScore using a local matrix: 11.81Score using the Hamming distance: 4Score for peptides with different length: 7Aligned sequence 1: WW-----SEVNR--AEFAligned sequence 2: --KTEEISEVN-IVAEFScore for peptides with non-canonical residues: 8.0


In the previous cases, we obtained single scores that depended on how the mutations were weighted. In a notebook in the repository, we showcase how to obtain a percentage similarity value using a single function wrapping the entire alignment.

Finally, we show a workflow to create a library following a set of suggestions based on specific mutations or random mutations in different positions of the initial peptide seed. The library will be internally named from mol1 to mol100. Then, the library is subjected to a similarity clustering analysis:from pepfunn.library import Librarylibtest=Library(population_size=100, mode="scanning", mode_scan="all", seeds=["FNCREWCWN"],pairs=[(1,"A"),(4,"L"),(6,"R"),(8,"M")], positions=[2,3,5,7], from_child=True,verbose=False)sequences=libtest.populationfrom pepfunn.clustering import simClusteringclust = simClustering(sequences=sequences)clust.run_clustering()centroids = clust.get_centroids()clust.plot_clusters(centroids)neighbors=clust.get_sim_reference("mol1")print(f"The top 5 closest neighbors of mol1 are:")for i,comp in enumerate(neighbors): print(comp)if i==5:break


The library is generated with 100 peptides using the sequence FNCREWCWN as a seed. Then, we assign specific pairs by including their position and the amino acid to add. We also activate an option in the function to add random mutations based on the given numbering. Finally, we activate a flag to randomly use any new peptides as a seed for the following suggestion to achieve a tree‐guided library design. With the final list of sequences, the following step is to run similarity clustering using Morgan fingerprints within the RDKit engine. We can generate a plot with the centroids and the number of molecules per cluster, and we can print the top 5 closest neighbors to a reference molecule in its cluster: The top 10 closest neighbors of mol1 in its cluster aremol19mol47mol6mol3mol100


### 3.3 Examples

To complement the overview of PepFuNN functionalities, two real examples are explained below. The code to reproduce the results is available in the repository.

#### 3.3.1 Clustering and Comparison of Cyclic Peptides

For this case scenario, we extracted from the ChEMBL database [37] a list of annotated head‐to‐tail cyclic peptides. The annotations include the HELM and BILN formats of the sequences that explicitly describe the head‐to‐tail amide bond, and the SMILES representations of the peptides. For this set, we filtered those having only natural amino acids, obtaining a list of 155 peptides for the clustering analysis.

**FIGURE 4:**
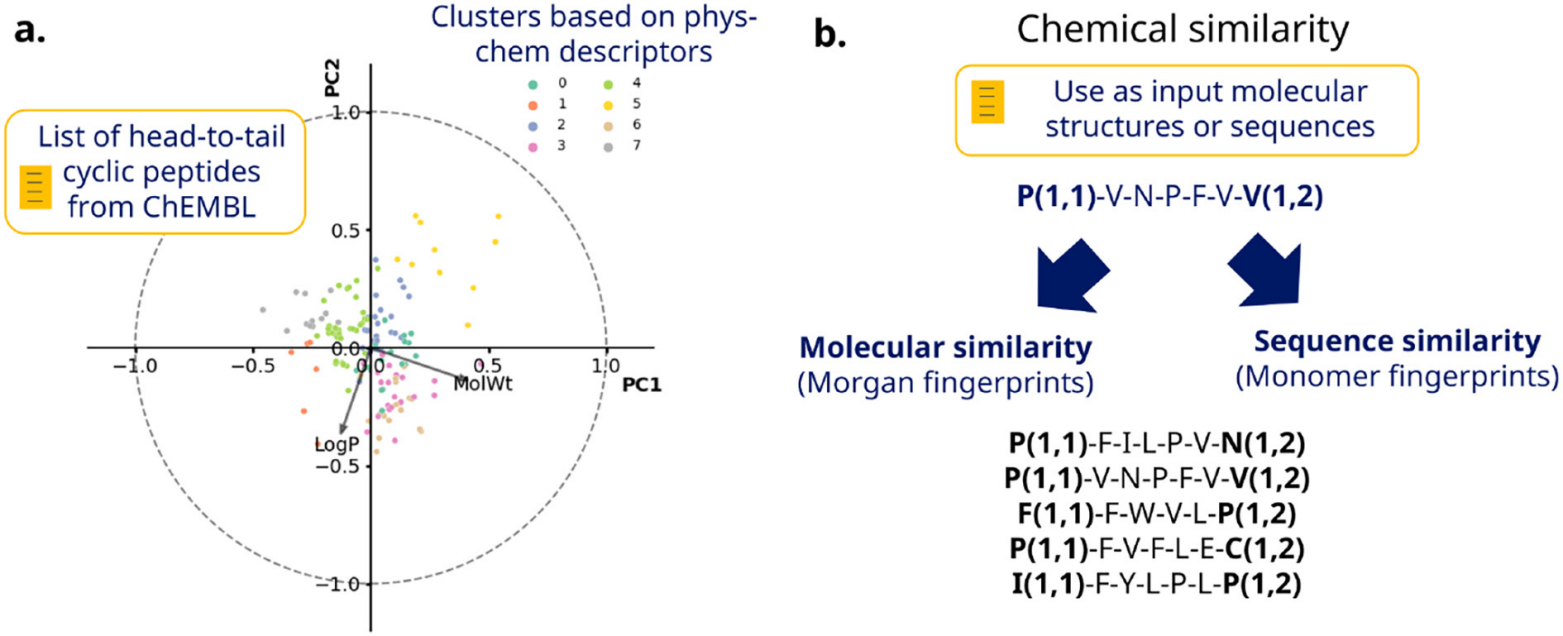
Strategy to cluster and compare cyclic head‐to‐tail peptides from the ChEMBL database. (a) The principal component analysis (PCA) plot includes arrows showing the importance of the main descriptors. (b) Chemical similarity strategy using as input Morgan fingerprints or monomer‐based fingerprints. The latest are obtained from the BILN representations of the peptides.

In the next step, we use RDKit‐generated Molfiles of the peptides from the SMILES. With this information, we call the *simClustering* function, where we provide the IDs, Molfiles, and sequences. Internally, the molecules are converted to Morgan fingerprints to check the similarity between a reference sequence and the remaining molecules. The Tanimoto coefficient captures the percentage similarity for each pair of molecules. As a parallel approach, we generated monomer‐based fingerprints based on the explanation in Section 3.1. For the latest, the similarity is also penalized by the order of the residues between the sequences. The analysis is complemented with a PCA plot of the properties using the *propClustering* function. The molecules are clustered with the K‐means algorithm using the descriptors. Figure 4 depicts the plot and a summary of the similarity strategies.

#### 3.3.2 Library Design

**FIGURE 5:**
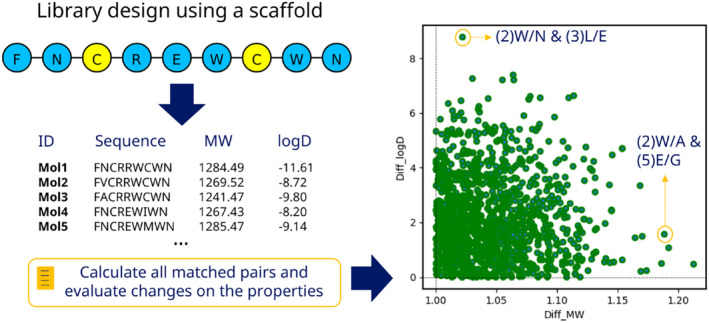
Example of a peptide library design and analysis using a sequence seed as reference. Based on a list of suggested mutations and positions, a library containing 100 sequences was generated, and a matched pairs analysis was run to observe the effect of specific mutations on two physicochemical properties: the molecular weight and the predicted log. The right plot shows the mutations' distributions after filtering by thresholds. Two mutation combinations are highlighted, showcasing situations where these changes can improve one property without drastically affecting the other, as an example of SAR analysis in a bioactivity assay.

To exemplify the analysis of peptide libraries, we used the *Library* function from PepFuNN to build a set of 100 sequences based on a peptide seed. The modifications can be generated based on random mutations, or from a list of suggested mutations that can be obtained from any experimental or computational analysis. For this example, we provide a list of artificial suggested mutations in positions 1, 4, 6, and 8, and activate the generation of random mutations in position 2, 3, 5, and 7, as shown in the last code snippet of Section 3.2. For each sequence in the library, we calculated two properties, the molecular weight using the Sequence module from PepFuNN, and the logD metric using a pre‐trained regression model (logd_predictor.py), available in the Practical Cheminformatics GitHub repository (GitHub link). The model was downloaded and used apart from the PepFuNN code base. With the table containing the sequences and their properties, a matched pairs analysis was run to check the impact on both properties by single modifications, or by taking into account up to three mutations at the same time. We calculated the ratio between the molecular weights and the arithmetic difference between the logD values to do the comparisons. Then, we filtered all pairs with positive differences. This filtering is helpful to understand how the minor changes drive specific properties or for SAR analysis to understand substitutions responsible for increasing the potency based on their properties or position within the peptide sequence. A summary of the strategy is shown in Figure 5.

The pipelines, tables and plots are available in the notebooks folder of the code repository.

## 4 Conclusion

PepFuNN is a versatile Python package that can be easily installed and tested in any Linux environment. Many of the functionalities aim to support SAR studies with peptides, involving the design of customized peptide libraries, clustering sequences, and analyzing how slight modifications can drive more significant changes in a property or assay of interest. The programmatic access to PepFuNN allows embedding their functionalities into massive analysis pipelines that can be focused on commercial or academic purposes. A set of tutorials with multiple examples are provided in the code repository, and we expect the research community to keep using the protocols to improve the open‐source community around this drug discovery modality.

## Author Contributions

R.O. created, reviewed, and implemented the code and wrote the documentation and the manuscript. K.D. reviewed the code and wrote the documentation and the manuscript.

## Conflicts of Interest

The authors declare no conflicts of interest.

## Data Availability

Project name: PepFuNN (version 1.0) Project home page: https://github.com/novonordisk‐research/pepfunn
Operating system(s): Linux Programming language: Python 3 Other requirements: RDKit 2023.03.3 or higher; Biopython 1.7.9 or higher. Additional packages are installed from PyPI based on the requirements list. License: MIT The code is available as a Github repository. All the sequences used in the manuscript are random or extracted from public datasets. Any questions about the implementation can be directed to the author's email accounts.
